# Corrigenda to “LUBAC-mediated linear ubiquitination: a crucial regulator of immune signaling”

**DOI:** 10.2183/pjab.97.012

**Published:** 2021-04-09

**Authors:** Kazuhiro IWAI

In this paper, the following should be corrected:

(page 121, Fig. 1)

Insert “This figure is reproduced from Iwai^96)^ with some modifications.” at the end of the legend of Fig. 1.

(page 123, Fig. 2)

Insert “This figure is reproduced from Iwai^96)^ with some modifications.” at the end of the legend of Fig. 2.

